# Prenatal and Postnatal Sonographic Confirmation of Congenital Absence of the Ductus Venosus in a Child with Noonan Syndrome

**DOI:** 10.1155/2017/3068178

**Published:** 2017-09-06

**Authors:** Christopher L. Newman, Matthew R. Wanner, Brandon P. Brown

**Affiliations:** ^1^Department of Anatomy and Cell Biology, Indiana University School of Medicine, Indianapolis, IN, USA; ^2^Department of Radiology, Riley Hospital for Children at Indiana University Health, Indianapolis, IN, USA; ^3^Department of Medical Humanities and Health Studies, Indiana University School of Liberal Arts, Indianapolis, IN, USA; ^4^Department of Philosophy, Indiana University School of Liberal Arts, Indianapolis, IN, USA

## Abstract

The ductus venosus serves as an important vascular pathway for intrauterine circulation. This case presents a description of an absent ductus venosus in a female patient with Noonan syndrome, including both prenatal and postnatal imaging of the anomaly. In the setting of the anomalous vascular connection, the umbilical vein courses inferiorly to the iliac vein in parallel configuration with the umbilical artery. This finding was suspected based on prenatal imaging and the case was brought to attention when placement of an umbilical catheter was thought to be malpositioned given its appearance on radiography. Ultrasound imaging confirmed the anomalous course. This is in keeping with prior descriptions in the literature of an association between Noonan syndrome and aberrant umbilical venous drainage. This case illustrates the need for awareness of this condition by the radiologist, allowing for identification on radiographs and the recommendation for further confirmatory imaging. Further, the case illustrates the value of paying particular attention to the fetal course of the umbilical vessels in patients with suspected Noonan syndrome, as this population is particularly at risk for anomalous vasculature.

## 1. Introduction

Along with the foramen ovale and the ductus arteriosus, the ductus venosus serves as a major vascular shunt in fetal circulation during intrauterine development. Derived from three pairs of embryonic veins (cardinal veins, omphalomesenteric or vitelline veins, and umbilical veins), it primarily serves to direct a large volume of oxygenated blood from the placenta, past the liver, and directly into the right atrium. In combination with the foramen ovale, this ensures that the majority of oxygenated blood is directed to the heart and brain of the developing fetus [1]. Prior case reports, largely within the pediatrics literature, have described absence of the ductus venosus and its implications on fetal development. While the exact prevalence is unknown, it has been associated with several other congenital abnormalities [2]. Here, a case is reported in a female with Noonan syndrome with both prenatal and postnatal sonographic confirmation of the venous abnormality.

## 2. Case Report

The patient was born prematurely (34 weeks and 1 day of gestation) at an outside hospital to a 29-year-old woman by elective cesarean section. The pregnancy had been complicated by preterm premature rupture of the membranes at 28 weeks and 2 days of gestation as well as prenatal diagnoses of cystic hygroma, pericardial and pleural effusions, left renal artery duplication, and bilateral ventricular hypertrophy with worsening myocardial function. This conglomeration of symptoms was later confirmed to result from Noonan syndrome. Specifically, this patient was found to have a* PTPN11* gene mutation (c.923A>C; p.N308T).

**Figure 1:**
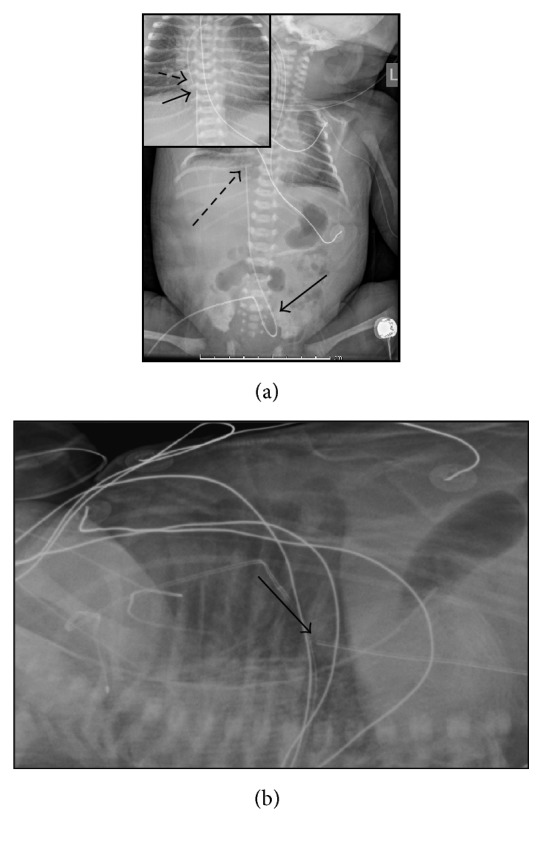
Anterior-posterior portable radiographs (a) and cross-table lateral radiograph (b) demonstrating the abnormal course of the umbilical catheter. The anterior-posterior view illustrates the caudal loop (solid arrow) as well as the superior ascent of the umbilical catheter to the right atrium (dashed arrow). The inset image of a subsequent anterior-posterior image reveals the close proximity of the umbilical catheter (solid arrow) and the peripherally inserted central venous catheter placed (dashed arrow) in the arm. The lateral radiograph reveals the position of the umbilical catheter (solid arrow) anterior to the expected position of the aorta.

At 12 hours of life, the patient became hypotensive and did not respond well to fluid resuscitation. Given the constellation of complications, the decision was made to transfer her to the tertiary care children's hospital for genetic assessment and cardiologic consultation. Upon arrival, the patient remained in respiratory distress and was hemodynamically unstable, and the placement of an umbilical catheter was attempted. A portable chest radiograph demonstrated a caudal loop of the catheter prior to ascending to the superior abdomen, so the catheter was assumed to be within an umbilical artery. However, the tip of the line was ultimately located to the right of the vertebral column (Figure 1(a)). Given this unusual position of the catheter, a cross-table lateral radiograph was performed. This demonstrated the tip of the umbilical line projecting over the expected location of the right atrium, at the level of the lower thoracic spine, and anteriorly and to the right of the expected position of the aorta (Figure 1(b)). A subsequent portable radiograph was obtained the following morning, redemonstrating the atypical position.

**Figure 2:**
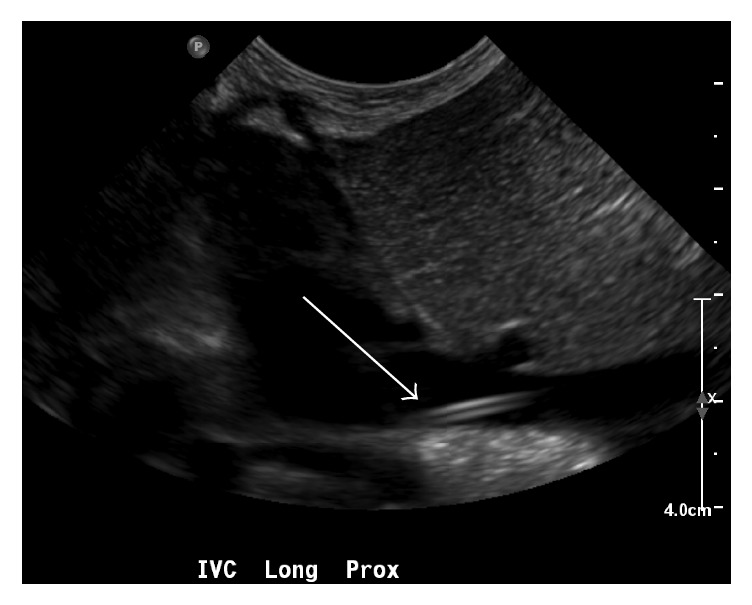
Sonographic imaging demonstrating the tip of the catheter (solid arrow) within the inferior vena cava (and not within the aorta). Also note that the size of the inferior vena cava is prominent.

An ultrasound with Doppler assessment was performed to accurately determine the positioning of the line. The aorta was found to be unremarkable and without an intraluminal catheter. Instead, the catheter was identified within the inferior vena cava, with the tip positioned at the lower cavoatrial junction (Figure 2). Confirming previous radiographs, the course of the catheter involved a caudal loop inferiorly and to the left, before coursing superiorly and to the right.

**Figure 3:**
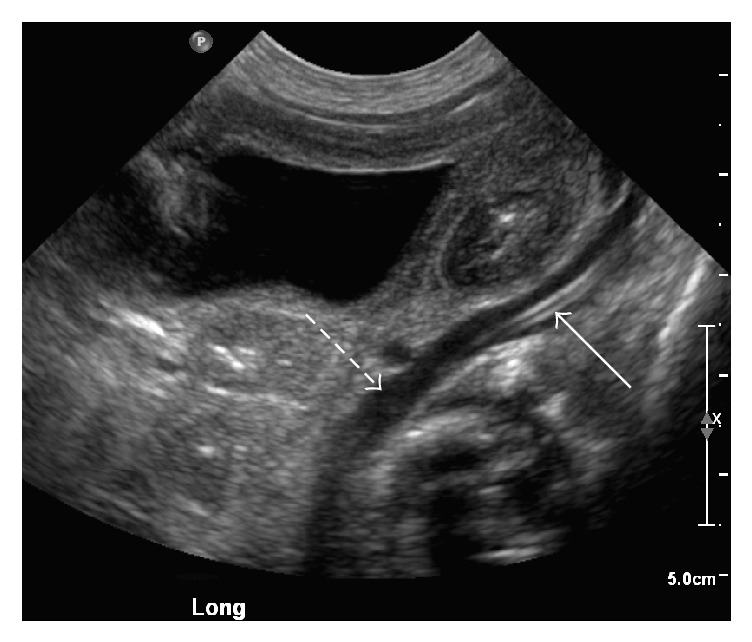
Sonographic imaging demonstrating the umbilical catheter (solid arrow) within the left iliac vein (dashed arrow) in close proximity to the bladder.

**Figure 4:**
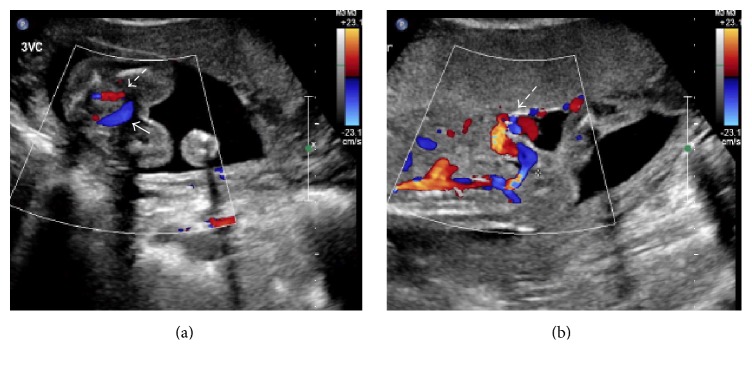
Transverse (a) and longitudinal (b) prenatal sonographic images demonstrating atypical umbilical venous anatomy at the level of the bladder. The transverse image demonstrates vessels coursing from the umbilicus into the fetus on either side of the bladder but with different Doppler flow directions indicating umbilical artery and vein. The longitudinal image demonstrates cord insertion and both arterial and venous flow into the fetal pelvis prior to joining the iliac vessels. Of note, there is no Doppler flow from cord insertion directly to the liver.

Further Doppler ultrasound imaging demonstrated an umbilical vein, which coursed inferiorly into the left iliac vein (Figure 3). Sonographic imaging was unable to identify the ductus venosus. The development of the portal system, however, was not assessed in this patient. The patient's obstetrical records were obtained from the outside healthcare system, and these indicated a suspicion of atypical umbilical venous anatomy seen during prenatal ultrasound (Figure 4). The umbilical catheter had no complications, and the remainder of the patient's hospital course was unremarkable. The patient is now one year old.

## 3. Discussion

Though several prior cases of agenesis of the ductus venosus have now been described [2], the underlying cause is still unclear. This lack of clarity is compounded by two factors. First, the prognosis of the anomaly is extraordinarily variable, ranging from otherwise healthy to intrauterine death. Second, the true incidence of the anomaly is likely underestimated since it is often only revealed by more detailed secondary sonographic examinations when suspicion for other congenital anomalies is high. Nevertheless, the specific course of the venous variability is an important prognostic factor [3]. Those with extrahepatic drainage of the umbilical vein (e.g., right atrium, inferior vena cava, and iliac veins) generally have a much poorer prognosis than those with intrahepatic drainage (i.e., into the portal venous system).

The current case describes this anomaly in a child with Noonan syndrome. While the abnormality is known to be associated with other congenital findings, Noonan syndrome is among the most common syndromic associations described [4–7]. Of note, all described patients with Noonan syndrome and agenesis of the ductus venosus (including the current case) have had direct umbilical drainage into the iliac veins.

Given its association with a poor prognosis, the radiologist should be aware of this vascular anomaly and be able to delineate the anatomy of the umbilical vessels in the fetus on prenatal imaging. This is especially true for those patients with prenatally diagnosed Noonan syndrome, among other associated conditions. In the immediate neonatal period, relying solely on radiography to verify the position of an umbilical arterial catheter in these patients can be misleading, and the presence of a “caudal loop” seen on radiograph should not be the sole determinant in confirming proper course of umbilical arterial line placement [8]. Any uncertainty in determining position of the catheter based on radiography should be verified with further imaging. Ultrasonography with Doppler assessment may be especially useful in verifying the positioning of an umbilical catheter and demonstrating vascular anatomy.
